# The Future Positive micro-intervention protocol: A program aiming to increase a healthy life-style among employees with a low socio-economic position

**DOI:** 10.3389/fpubh.2022.832447

**Published:** 2022-09-23

**Authors:** Karen Schelleman-Offermans, Robert A. C. Ruiter, Karlijn Massar

**Affiliations:** Department of Work and Social Psychology, Maastricht University, Maastricht, Netherlands

**Keywords:** health behaviors, low socio-economic position, future positive micro-intervention, intervention mapping, health inequalities

## Abstract

This paper describes the development of a Dutch micro-intervention, *Future Positive*, that aims to increase health behaviors among employees with a low socio-economic position (SEP), with the ultimate aim to decrease socio-economic health inequalities. Intervention Mapping (IM) was used to adapt previously developed psychological capital interventions into a micro-intervention suitable to be delivered in the work context for employees with a low socio-economic position. The first 4 steps of IM including the results of pre-testing the developed intervention program are described. Step 1 consists of the needs assessment, and investigated (a) the individual determinants of health behavior and health inequalities, and (b) the needs of employees with a low SEP and their employers regarding the implementation of the intervention at the worksite. Matrices-of-change were produced in Step 2, and relevant methods and applications were selected in step 3. Step 4 involved the intervention development, resulting in a brief micro-intervention that will be delivered in small groups, guided by trained facilitators using motivational interviewing techniques. Program materials include informative video-clips and active and cooperative learning exercises. The intervention was pre-tested among three groups of employees. The IM process, as well as the pre-testing, revealed that emphasizing autonomy and using easy to understand and mostly visual materials offered in chunks is essential for a well-tailored intervention that is suitable for people with low SEP. Also, participation should be facilitated by employers: It should be free of costs, offered during working hours, and take place at the job site. Results showed that the Future Positive micro-intervention is substantiated by theory, applicable in a work setting (high reach), and tailored to the needs of employees with a low SEP. We therefore fill the gap in this existing range of interventions aimed to improve life-style behaviors and contribute to theory-based interventions aimed to decrease the SEP-Health gradient.

## Introduction

Education, income and wealth determine largely what resources people hold that determine health behaviors and outcomes (1–3). Large socio-economic health disparities exist even in high-income countries such as the Netherlands, where people with a low education [a proxy for a low socio-economic position (SEP, i.e., low education and/or income)] on average live 7 years shorter and spend 19 years of their lives with a lower quality of life than higher educated people (1). In addition, people with a low SEP more often show an unhealthy life-style (2), which is related to various types of diseases in the long term (1) and is responsible for almost 20 percent of the disease burden, 35 thousand deaths, and 9 billion euros in health care expenditure in the Netherlands (3).

Smoking and obesity are the main preventable causes of mortality and death in the Netherlands (1) and these life-style related behaviors are more prevalent among lower SEP individuals, who often are employed in low-paid jobs that do not require a higher education (2). It follows that health promotion efforts targeting these lifestyle-related behaviors among this group may decrease socio-economic health disparities. However, individuals with a low SEP are a difficult to reach group (4), and the intervention materials used in most existing life-style enhancement programs are insufficiently suitable for people with a low SEP (5). There is therefore a need for intervention programs targeting people with a low SEP that use tailored intervention materials and are offered in a way that is suitable for this vulnerable group, and are offered in a context where these individuals can be reached. Implementing such an intervention in the work setting—or more specifically, the job site—may facilitate the access to large groups of individuals with a low SEP (6, 7).

**Figure 1:**
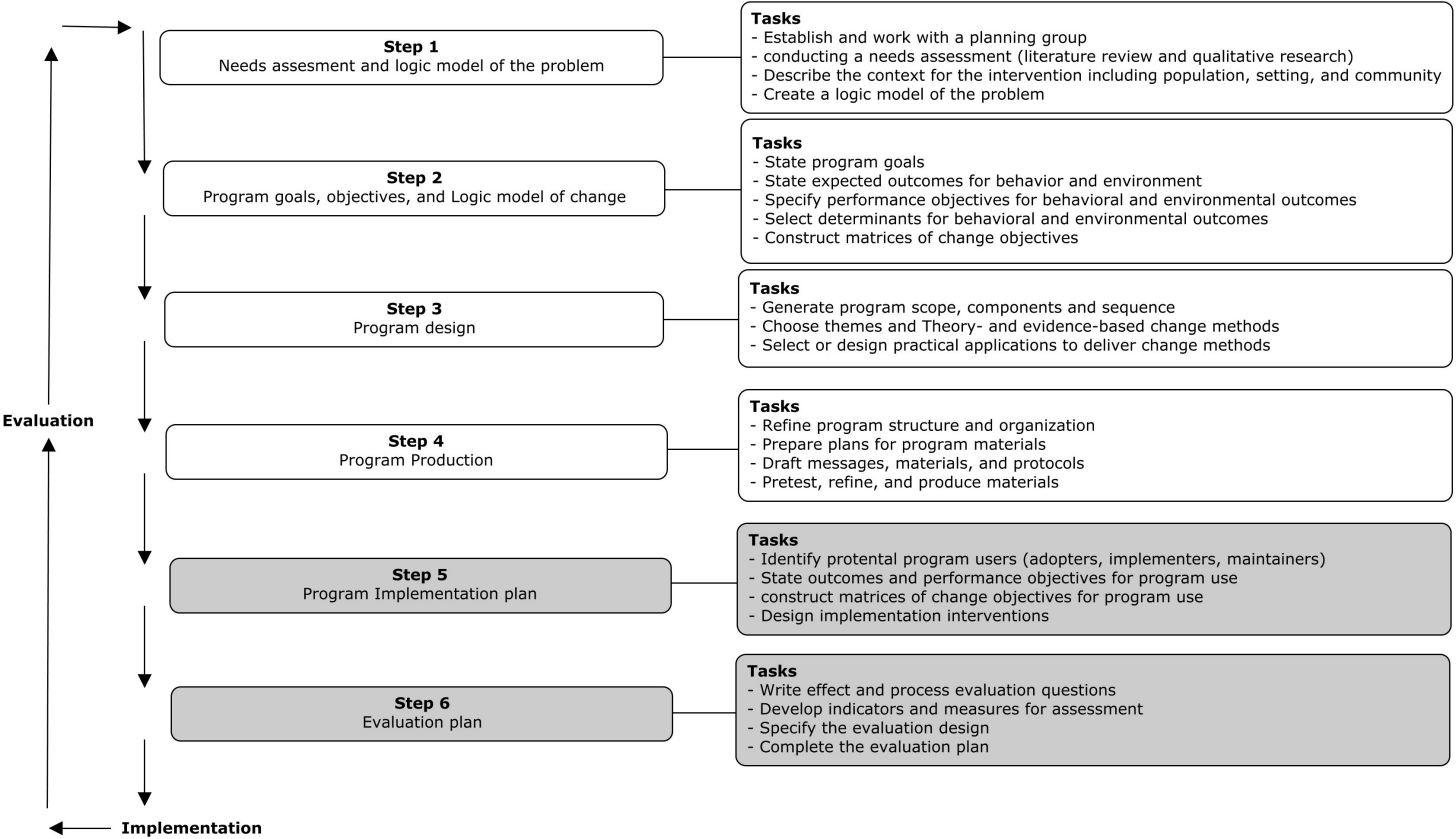
Intervention mapping steps as adapted from Bartholomew et al. (8).

This paper provides a detailed description of the development of the *Future Positive* micro-intervention protocol; a program aimed to increase life-style related health behavior of employees with a low SEP consisting of two intervention sessions of 2 h and a 12 week after-care period. To tailor the intervention to the target group and organization-specific contextual factors, Intervention Mapping (IM) was used (8). IM is a stepwise participatory methodology for systematically developing, implementing, and evaluating health-promotion programs or adapting already existing interventions to specific target populations and contexts. IM is characterized by an iterative or formative process, meaning that the output of each step is used as input for the subsequent step, and for the adjustment of already made decisions in previous steps (see Figure 1 for an overview) (8). The participatory methods used aim to actively involve stakeholders (e.g., target group, implementers, and adopters) using shared-decision making processes to accommodate their perspectives, and to develop a sense of ownership and commitment to change—both among the target group as well as the larger organizational context. Thus, IM ensures that a bottom-up approach is used in which there is a central role for the proposed target population of the intervention.

The main aim of this study is to provide other researchers and professionals with a theory-based description of the program development (step 1–4 of IM) of the Future Positive micro-intervention. Our main research questions during the first four steps of IM were: What are the needs of employees with a low SEP when it comes to their lifestyle-related behaviors; What should be changed to increase the lifestyle-related health behaviors of low SEP employees; How (what delivery context, mode and form) can the intervention be tailored to employees with a low SEP to effectively change their lifestyle related behavior. The Future Positive micro-intervention protocol has been specifically developed to be delivered during work hours and on the job sites of employees in low-paid jobs that do not require a higher education (i.e., low SEP) with the aim to increase their life-style related health behavior. This intervention, therefore, fills the gap in the existing range of (workplace) interventions aimed to improve life-style behaviors, and specifically, addresses the need for theory-based interventions to decrease socio-economic health inequalities. In the Methods section, we will briefly explain the tasks that were conducted in the IM steps. Thereafter, the results for each of these IM steps will be presented and a discussion and reflection of these results are presented in the Discussion section.

## Materials and methods

### Intervention mapping (IM) approach

It goes beyond the aim of the current study to describe all 6 steps of the IM process (8) including the development of an adaption and implementation plan (step 5) and evaluation plan (step 6) (see Figure 1 for an overview of all steps). Therefore, we will focus on the first four steps of IM. For an extensive description of IM, see Bartholomew et al. (8). The protocol to develop this intervention including its data collection method was approved by the Ethical Review Committee of Psychology and Neuroscience (Reference Number: 198_13_09_2018). Informed consent was obtained for all participants in the interviews, focus groups, and the pre-testing. The development of the Future Positive intervention has been conducted in co-creation with a company in the cleaning industry.

#### Step 1: Needs assessment and logic model of the problem

Tasks that were completed in the first step included establishing and working with a planning group for the complete IM process and conducting a needs assessment. The formation of a planning group that consists of researchers, members of the target population, and other relevant stakeholders formalizes the participatory nature of IM. Moreover, a planning group is essential to ensure, among other things, the development of logistically and culturally appropriate interventions and to enhance recruitment capacity.

Gaining insight into the needs of the target population with respect to their health and how the intervention should be delivered, the target populations' health problems, and the etiology of these health problems, as well as the perceptions about these issues of other important stakeholders (e.g., managers or foremen) were the focus in the needs assessment. A mixed-methods approach was used that included a literature review and collecting additional qualitative data from employees working at the company in the cleaning industry. We reviewed the literature regarding the etiology of health problems of people with a low SEP, the effectiveness of workplace interventions, and literature on the theoretical methods and practical applications that already have shown the ability to effectively change the (health) behaviors of the target group [e.g., (9, 10)]. Additional data were collected by conducting two focus-group discussions (Total *n* = 9, 55.6% female, age ranges from 42 to 65 years) among frontline employees (1 h), and five in-depth interviews (1.5 h each; one management board representative, one regional manager, one object manager, one direct supervisor, and two occupational health professionals). Themes that were discussed in the focus-group discussions and interviews were (a) which type of life-style behaviors and health issues are most prevalent among frontline employees with a low SEP; (b) perceptions about which factors (determinants) are associated with these lifestyle behaviors, (c) perceptions about willingness to change (e.g., the degree to which foremen thought frontline employees would we willing or motivated to change their life-style related behavior); (d) what type of intervention materials (e.g., video-clips, visuals, booklets) may be suitable for front-line employees, (e) perceptions about the preferred delivery method (e.g., materials, exercises, group composition, etc.); (f) what facilitating or hindering factors could play a role when trying to recruit (and retain) the target group to participate in an intervention, and (g) how to implement such an intervention at their worksite. Interviews and focus-group discussions were transcribed and thematically coded within the themes (see a–f above) that were discussed in the focus groups and interviews using Atlas-ti 8.4 (11). Moreover, a brainstorm session with the planning group was conducted in which the results of the needs assessment were presented and discussed for a member-check. The last task in this step is to combine the results of the qualitative exploration with evidence from the literature review to construct a logic model of the problem.

#### Step 2: Program goals, objectives, and logic model of change

In the second step, it is determined what the developed intervention aims to change, and for whom. Specific tasks consisted of specifying expected intervention outcomes, so-called program goals, and determining specific performance objectives (PO's) at the sub-behavioral level that indicate how to reach the program goals. Also, significant and changeable determinants are chosen for each PO that needs to change in order to perform the PO. Then, matrices of change were developed that connect the PO's and determinants by including the social-cognitive and/or affective desired changes (Change Objectives, COs) to reach the program goals. All objectives (both the POs and COs) are developed using the results of Step 1, and are formulated in a SMART (Specific, Measurable, Attainable, Realistic, Timely) way.

#### Step 3: Intervention design, selection of theory-based methods, and applications

In the third step, the previous literature analyzed for the needs assessment (step 1) was used to select theory-based methods that were translated into context-specific practical applications. A taxonomy of behavior change methods was used, developed by Kok et al. (12), to select the appropriate methods for each formulated change objective related to the specific targeted determinants, respecting their parameters for use (conditions under which the specific methods are effective). For the determinants that were not present in this taxonomy, but showed to be of importance in explaining socio-economic health inequalities from the results of the needs assessment (step 1), change methods were chosen based on the effectiveness of change methods published in previous studies. Available program materials from previously developed interventions were reviewed for potential use, and new materials were developed in line with the previously specified objectives as well as theoretical parameters for use (13). All proposed methods and applications were discussed with the planning group.

The last task in this step consisted of creating Acyclic Behavior Change Diagrams for each specified performance objective using the *ABCD shiny app* (14), which are visual representations of the causal (i.e., what influences what) and structural (i.e., what consists of what) assumptions underlying the behavior change intervention.

#### Step 4: Program development and production

The selected theory-based methods and practical applications from Step 3 were combined to develop the final intervention program, including topics for presenting examples to participants (e.g., about quitting smoking), sequence and tailored materials. All developed materials were presented to the planning group and their feedback was processed to ensure that the developed intervention materials are in line with the needs of the target population and the company. Then, the intervention was pre-tested among three groups of employees with a low SEP (total *n* = 10) at two different companies (in the cleaning and steel production industry) and focus-group discussions among these participants were conducted and reflections of the facilitator were used to evaluate the actual implementation, inhibiting and facilitating factors observed by the facilitator, and the experiences of participants with the intervention.

## Results

### Needs assessment (step 1)

#### Literature review

Previous research has shown that people with a low SEP more often show an unhealthy life-style than people with a higher SEP (2). In an attempt to explain these socioeconomic differences, the Reserve Capacity Model (RCM) (15–17) explicates the mediating role of “reserves” in the SEP-health gradient. The RCM posits that, compared with people with a higher SEP and due to worse social living conditions, individuals with a low SEP experience more daily hassles and major stressors in their lives, and simultaneously have fewer “reserves” (e.g., psychological capital, social support networks) to cope with these stressors. According to the RCM, these higher stressors and fewer reserves have direct and indirect effects on lifestyle behaviors *via* negative cognitions and/or emotions such as a higher present-fatalistic time perspective (15–17), which is associated with feelings of hopelessness and a lack of control over life (18, 19). Individuals with a low SEP indeed hold beliefs that health outcomes are the result of predetermination and therefore inevitable and think less about the future than higher individuals with a higher SEP (19). One's time perspective describes how one's subconscious perception or weighing of the past, present, and future influences decision-making, including health-related decision making (19, 20). Indeed, time perspectives have consistently been linked to health behaviors, such that a future-oriented perspective is associated with increased protective health behaviors (20) and causes the individual to regulate their behavior, establish goals and expectations, and to motivate and monitor performance. Conversely, there is evidence that fatalistic beliefs about disease prevention (present fatalistic life perspective) are associated with less weekly exercise, smoking, eating less than the recommended five portions of fruit and vegetables per day and making less healthy behavioral choices (e.g., avoiding cancer screenings) (21, 22). These findings imply that, in order to increase the life-style related behaviors of people with a low SEP it is important to increase their reserve capacities, and shift their time perspective to more future-oriented thinking. We identified two variables that may accomplish this: *psychological capital* (PsyCap) and *social support*.

The intra-personal reserve PsyCap consists of four psychological constructs (hope, optimism, resilience, and efficacy) and has shown to influence self-rated health and health conditions in a positive way (23). Although the separate constructs each have unique contributions to an increase in healthy behaviors, they also share a common core, which is characterized by a focus on identifying one's strengths, making positive appraisals of one's chances of success, and having a perception that one's goals are within reach and under one's control. Briefly, Luthans and Youssef-Morgan (24) define PsyCap as an “(…) individual's positive psychological state of development, characterized by: (1) having confidence and skills to take on and put in the necessary effort to succeed at challenging tasks (efficacy); (2) making positive attributions about succeeding now and in the future (optimism); (3) persevering toward goals and, when necessary, redirecting paths to goals in order to succeed (hope); and (4) when beset by problems and adversity, sustaining and bouncing back and even beyond to attain success (resiliency).” Health PsyCap can thus be seen as drawing from one's psychological resources of hope, efficacy, resilience, and optimism in making positive appraisals of one's health-related circumstances and the probability for health-related success, based on motivated effort and perseverance (25). High levels of PsyCap have shown to have a positive association with wellbeing and health outcomes such as lower body mass index (BMI) and cholesterol levels (26). Also, PsyCap has shown to mediate the link between a low socio-economic position on the one hand, and health outcomes (23) and health behaviors (27) on the other hand, illustrating the possible buffering effect PsyCap has.

Importantly, PsyCap has shown to be open to development and can be developed in multiple domains (24–26), among which the health domain. Individuals participating in health PsyCap interventions (PCI) set personally meaningful and attainable health end-goals that are divided into several sub-goals. Once sub-goals are attained, the resulting motivation, commitment, and satisfaction of achieving one's health goals are expected to result in increased motivation, hope, and optimism for reaching additional goals. Moreover, PCI use goal-setting, chunking, goal visualization, and cooperative learning as change methods that have shown to be able to increase PsyCap in brief group interventions, resulting in sustainable (up to 7 months) behavioral change (28).

Social support has been defined as emotional, instrumental, and informational aid or appraisal exchanged through social interactions (29, 30). Social support, an interpersonal reserve, has shown to increase health and wellbeing and decrease morbidity and mortality rates (30, 31), directly through increasing health behaviors such as physical activity, and indirectly by buffering against adverse effects of stressors on health (29–31). Changing levels of social support is possible—however, the evidence of the effects of increasing levels of social support (e.g., implementing buddy systems) on health behaviors such as smoking cessation is scarce (32). On the other hand, strong evidence for a direct positive effect of social support on wellbeing, by strengthening morale or providing a sense of connectedness and being cared for, has been shown in several worksite studies (33, 34). Importantly, behavior change is most likely when an individual is *motivated* to show healthy behaviors. Especially motivations that are more autonomous—i.e., engaging in behaviors for self-endorsed (intrinsic) reasons—result in better health outcomes, increased wellbeing, and increased likelihood of behavioral adoption and maintenance (35). To facilitate a more autonomous motivation three basic needs should be satisfied; autonomy (perceptions of empowerment and having a choice), competence (feelings of efficacy) and relatedness (feeling close to and valued by others) (36). As the brief literature review above suggests, these needs could be met if psychological capital and social support are present in sufficient amounts. Moreover, it is possible to increase people's autonomous motivation toward behavioral change. For instance, motivational interviewing (MI) has shown to positively affect behavior, even after brief one-off sessions. A meta-analysis of interventions using MI in health-related contexts (e.g., weight reduction, alcohol and smoking cessation) revealed that the technique was more effective than advice giving in improving behavioral (e.g., number of cigarettes, alcohol consumption) and health-related (e.g., BMI, cholesterol) outcomes (37, 38). Further, Hardcastle et al. (39) showed positive effects of MI in a disadvantaged community, such that the participants increased their physical activity levels and family social support.

#### Themes that resulted from the qualitative data describing the context for the intervention

##### Setting

Results from the interviews and focus groups showed that companies with a large proportion of employees with a low SEP work with protocols to ensure efficient production rates and work in shifts (some even scatter different shifts over 1 day, e.g., 6:00–9:00 and 16:00–20:00). Regarding the physical work place it can be the case that employees work at different locations of customers (e.g., in cleaning companies), however, for production companies, employees normally do work in one location (e.g., a factory). The production in assembly line work is however mostly dependent on the production of other employees which means that breaks and working hours need to be aligned.

##### Health behaviors of employees with a low SEP and related factors

The organizational stakeholders (in-depth interviews) indicated that their employees generally had high absenteeism due to illness, and that a large proportion of the employees aged 45+ showed chronic health conditions, such as diabetes, COPD, or overweight. Organizational stakeholders indicated that many of their employees have financial problems, and these, combined with a low income may make it difficult for the target group to buy healthy food or to join a sports club due to these often being (too) expensive. The stakeholders also indicated they perceived the intrinsic motivation of their employees to change their unhealthy behaviors as low. Considering the type of unhealthy behaviors, the results indicated that, although all health behaviors (e.g., eating, exercising, sleep, substance use) most likely can be improved among this specific group, smoking was mentioned both, by the employees themselves as well as by the organizational stakeholders, as the most prevalent and important behavior to change. Results of the focus group interviews indicated that working in shifts negatively affected their life-style, in particular their eating and sleeping habits.

##### Suggestions for methods and applications

Some important issues emerged from the interviews with the organizational stakeholders and the focus groups with the employees regarding the intervention. For example, stakeholders emphasized that for participants to be highly involved in the group discussions, creating a safe environment in the first session was deemed necessary. Given the possibly limited cognitive level of comprehension and the possibly limited language processing skills of employees, it was indicated in the interviews that it is important that all the information—both instructions by the facilitator as well as the participant materials—should be easily comprehensible, and should focus on visual information rather than written text. In contrast, the employees themselves mentioned that a booklet in which they could write down their achievements and reached (sub) goals, could help them stay motivated toward their end goal. They further recommended that the information should be delivered step by step, in small chunks, and should offer a variety in working methods, to facilitate information processing and sustained attention among the employees.

Results of the focus group discussions with employees indicated that they positively evaluated the fact that the intervention was group-based. In their view, helping each other in the group setting could really stimulate them in forming and achieving health-related goals. They indicated that the option of bringing a family member of friend to the sessions could enhance their sense of psychological safety and social support; not only during the sessions but throughout the entire intervention time path. All respondents indicated that small groups (no more than 5 or 6 participants) were preferred, without the presence of a foreman or manager, and ideally would focus on a shared health behavior the employees wanted to change (e.g., a “stop smoking” group, or a “lose weight” group).

##### Possible facilitating and hindering factors regarding recruitment and implementation

Three factors were regarded to be important to increase the willingness of employees with a low SEP to participate in an intervention to increase health behaviors. First, providing concrete, easy to understand, and accessible information about the intervention, stressing the autonomy of participants to change their health behavior and in which way, was seen as a necessity to increase motivation to participation. Secondly, when recruiting participants, an internal ambassador (e.g., a supervisor or other employee, someone they already know and trust) promoting participation in the intervention would help to increase participation. Last, all respondents mentioned that participation in the intervention during (paid) working hours was a pre-condition.

The employees usually had to work at different locations during the week (inherent to the cleaning industry), which the respondents mentioned as a challenge for the efficient implementation of the intervention. If the intervention would, for example, be offered at the organization's headquarters, this would pose a challenge for participants without easy access to transport. Intervention activities should therefore, as much as possible, take place at the actual job sites. Permission to use the actual job site (a building owned/hired by their customers) to deliver the intervention was needed. Furthermore, since most companies with a high number of low-SEP employees suffer from staff shortages, the organizational stakeholders mentioned that it might be difficult to find replacement workers for employees participating during working hours.

#### Logic model of the problem

**Figure 2:**
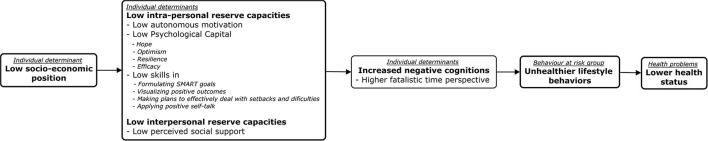
Logic model of the problem. Based on the Reserve Capacity Model [e.g., (15)].

To summarize, determinants of socio-economic health inequalities are selected based on the RCM model (15–17), with a focus on individual determinants. Studies that have tested the RCM indicate that the reserve capacities PsyCap (intrapersonal) and social support (interpersonal) as well as time perspectives (cognitions) are associated with health behavior, especially for people with a low SEP [e.g., (24)]. Also, the degree to which someone is autonomously motivated to change his or her health behaviors is important for sustained behavior change (40). The results from the interviews and focus group discussions underline the importance of a high autonomic motivation, social support, and intrapersonal reserves PsyCap and skills. Informed by the results of the needs assessment, an overview is given of the logic model of the problem used in this intervention in Figure 2.

### Program goals and matrices of change objectives (step 2)

#### Program goals

Considering that companies with a large proportion of employees with a low SEP often experience a shortage in personnel, with employees working in shifts and sometimes working at different (customers') locations, implementing an intervention during working hours can be logistically challenging. To increase the willingness of companies and participants to implement or participate in the intervention and decrease the burden on their time, we opted a priori for a micro-intervention. This micro-intervention included only two intervention sessions of 2 h each guided by trained facilitators, followed by a 12 week after-care period in which participants are remotely supported. Furthermore, due to reasons of feasibility, we have opted for an individual approach, targeting employees with a low SEP, and do not include higher levels of influence (and its agents) in the intervention.

The main program goal of the Future Positive micro-intervention is to increase (at least) one self-chosen aspect of the life-style behavior of employees with a low SEP during a period of 12 weeks. For example, the employee could focus on eating more healthily, exercising more, improving sleeping, decreasing or quitting smoking, or reducing alcohol or other substance use. From the logic model of the problem (Figure 2) it can be derived that to increase the health behavior of individuals with a low SEP, it is important to increase their autonomous motivation, their intra- and interpersonal reserves such as psychological capital (consisting of hope, efficacy, resilience, and optimism) and social support (16–18), stimulate perceptions and cognitions of a positive future. We propose that targeting the determinants presented in Figure 2 should provide employees with a low SEP with the proper pre-conditions, together with the appropriate skills, to perceive one's health as being under one's own control, now and in the future, enabling them to achieve their health-related goals.

#### Matrices of change objectives

Seven specific *Performance Objectives* (POs) and related *Change Objectives* (COs) for participants were determined for these specified changeable determinants (see Table 1 for an overview). For example, the first Performance Objective (PO1) was “*Employee with a low SEP clearly defines his motivation to improve his life-style behavior*.” In order to reach PO1, the determinants intention to change health behavior (autonomous motivation), future-oriented positive cognitions (optimism and future time perspective) and perceived social support need to be increased. Several Change Objectives were formulated to accomplish change in these specific determinants. For example, participants were stimulated to demonstrate more reasons for changing than for not changing (CO AM.1b; Table 1), and make more positive attributions about the benefits of changing than not changing (CO O.1a; Table 1).

**Table 1:** Performance objectives (POs), determinants, and change objectives (COs) in the Future Positive micro-intervention.

	**Change objectives (COs) for each relevant determinant**						
	**Future-oriented positive cognitions and emotions**			**Goal-oriented cognitions and skills**		**Norms**	**Intention to change**
	**Future time perspective**	**Psychological capital**				**Social support**	**Autonomous motivation**
**Performance objectives (POs)**		**Hope**	**Optimism**	**Resilience**	**Efficacy**		
PO1: Employee with a low SEP clearly defines his motivation to improve his life-style behavior	TP. 1a: Believe in the value of future health	N.A.	O.1a: Make more positive attributions about the benefits of changing than not changing	N.A.	N.A.	S.1a: Believe that friends, colleagues, employers and/or family will support reasons to improve health behavior	AM.1a: Express the need to change health behavior AM.1b: Demonstrate more reasons for changing health behavior (setting goals) than not changing behavior. AM.1c: Express trust- and change-language
PO2: Employee with a low SEP sets at least 1 specific and achievable end goal to improve their life-style	N.A	H.2a: Believe that setting SMART end goal will support capability to change health behavior in a sustainable way	O.2a: Make positive attributions about reaching the end goal and thereby improving future health O.2b: Express positive expectations about goal realization	N.A.	E.2a: Express confidence in own ability to achieve end goal. E.2b: Demonstrate setting a SMART end-goal	S.2a: Believe that friends, colleagues, employers, and/or family will support the decision to improve health behavior	AM.2a: Show willingness to set goals
PO3: Employee with a low SEP divides the end-goal in at least 3 achievable and specific sub goals	N.A.	H.3a: Believe that setting SMART sub goals will support capability to change health behavior in a sustainable way H.3b: Believe that dividing the end goal in several smaller steps will support capability to change health behavior in a sustainable way H.3c: Believe to remain positive about persevering toward goals when dividing the end goal in several smaller steps	O.3a: Make positive attributions about reaching the end goal in several smaller steps O.3b: Express positive expectations about goal realization	N.A.	E.3a: Express confidence in own ability to reach sub goals E.3b: Demonstrate setting SMART sub-goals	S.3a: Believe that friends, colleagues, employers, and/or family will support the decision to improve health behavior	AM.3a: Show willingness to set goals
PO4: Employee with a low SEP visualizes how end goal can be reached and experiences and mentions (future) mental successes	TP.4a: Aware that future health can be reached	H.4a: Believe to remain capable in persevering toward goals when experiencing the positive cognitions/emotions of (mental) success and overcoming potential difficult situations by visualizing end goal	O.4a: Make positive attributions about experiencing mental successes and overcoming potential difficult situation by visualizing end goal	R.4a: Express confidence to bounce back when encountering difficulties on the way to goal(s)	E.4a: Demonstrate visualizing reaching goal(s) E.4b: Express confidence to reach goal(s) E.4c: Write down how future successes will be celebrated, even small successes	S.4a: Feel supported in the ability to reach goal(s) by learning from (previous) experienced successes of others in the group	N.A.
PO5: Employee with a low SEP makes an inventory of obstacles and difficult situations he may encounter on the way to his goal(s)	TP.5a: Aware that putting effort in living more healthily now will result in better future health	H.5a: Expect that making an inventory of all the obstacles and difficult situations on the way to the goal(s) will increase perseverance toward goal(s)	O.5a: Make positive attributions about the effectiveness of making an inventory of obstacles/difficult situations to reach goal(s)	R.5a: Express confidence to identify all relevant difficulties and obstacles on the way to their plan	E.5a: Express confidence in the ability to make an inventory of the obstacles and difficult situations E.5b: Describe difficult situations and obstacles on the way to goal(s)	S.5a: Feel supported by others to identify all relevant obstacles and difficult situations	N.A.
PO6: Employee with a low SEP formulates plans to overcome obstacles and difficult situations he may encounter on the way to his goal(s)	TP.6a: Aware that putting effort in living more healthily now will result in better future health	H.6a: Expect capability in perseverance toward goal(s) H.6b: Describes redirecting pathways in the case of setbacks H.6c: Feel in charge H.6d: Expect that monitoring progress and celebrating small successes will help increase capability to persevere toward goal(s)	O.6a: Make positive attributions about the effectiveness of self-defined plans to avoid or overcome obstacles/difficult situations to reach goal(s) O.6b: Make positive attributions toward using positive self-talk when encountering set-backs	R.6a: Express confidence to overcome difficulties and bounce back when encountering difficulties in reaching goal(s)	E.6a: Express confidence in the ability to make plans to overcome or prevent difficult situations or obstacles on the way to goal(s) E.6b: Demonstrate making plans to avoid obstacles and difficult situations on the way to goal(s) E.6c: Demonstrate making if.. than.. plans to overcome obstacles and difficult situations on the way to goal(s)	S.6a: Feel supported by others to make effective plans to overcome setbacks or avoid difficult situations	N.A.
PO7: Employee with a low SEP adheres to the personally formulated plans to overcome or avoid obstacles and difficult situations on the way to the goal(s), monitors progress toward their goal(s) and celebrates successes of reached goals	TP.7a: Aware that putting effort in living more healthy now will result in better future health	H.7a: Feels in charge of own health	O.7a: Make positive attributions about the effectiveness of monitoring progress and celebrating reached successes on the way to the goal(s) O.7b: Make positive attributions about succeeding now and in the future	R.7a: Express confidence to overcome difficulties and bounce back when encountering difficulties in reaching goal(s) R.7b: Demonstrate overcoming or avoiding difficulties and obstacles on the way to goal(s) by adhering to plans and using positive self-talk	E.7a: Express confidence in the ability to monitor progress toward goal E.7b: Demonstrate monitoring the progress toward goal E.7c: Express confidence in ability to reach sub- and end goals E.7d: Demonstrate reaching (sub)goal(s)	S.7a: Feel supported by others to overcome setbacks and persevering toward end goal	N.A.

We have not included separate performance objectives (PO) targeting the fourth intervention module: social learning and active use of social support, but opted for a small group setting. Research shows that interventions aimed at behavioral change delivered in groups are more effective than individual interventions (37). In addition, we encourage participants to actively involve family members or friends in their behavioral change, either by taking them to the workshops and/or by involving them in their action plans.

### Theory- and evidence-based methods and practical applications (step 3)

#### Intervention scope, components, and sequence

**Figure 3:**
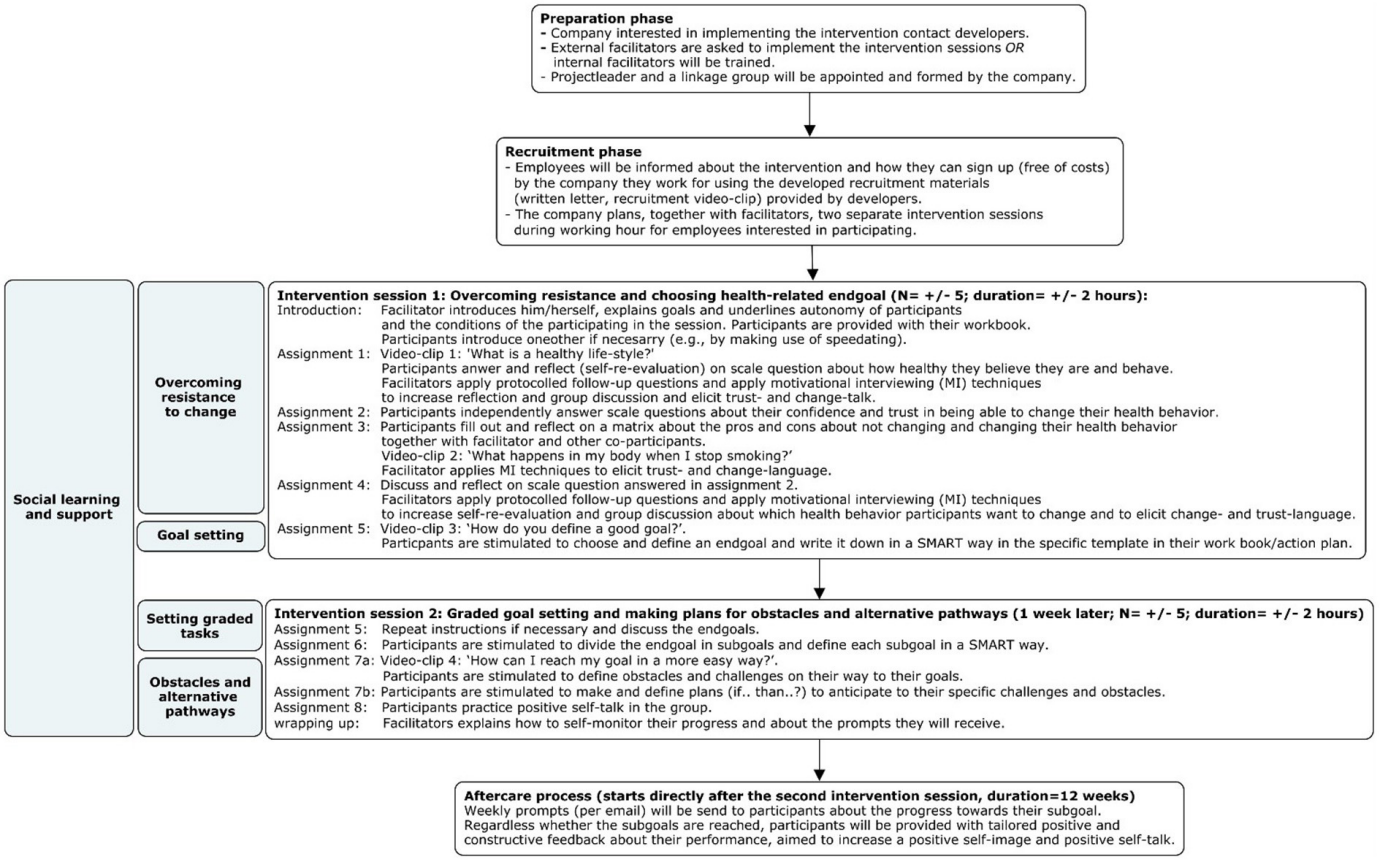
Overview of intervention activities.

The four basic intervention components of the Future Positive micro-intervention are: (1) overcoming resistance to change (targeting autonomous motivation), (2) goal setting and graded tasks (targeting future time perspective, hope, optimism and efficacy), (3) obstacles and alternative pathways (targeting resilience and efficacy), and (4) social learning and active use of support (increasing interpersonal reserves; perceived social support from relevant others). The sequence of intervention activities linked to the intervention components and practical applications are displayed in Figure 3.

#### Themes, theory-, and evidence-based change methods and practical implications for performance objectives

The Acyclic Behavioral Change Diagrams for each PO can be found in Supplementary materials. These diagrams show a visual representation for each PO of the causal (i.e., what influences what) and structural (i.e., what consists of what) assumptions underlying the behavior change intervention (13).

To realize PO1 (*Employee with a low SEP clearly defines their motivation to improve their life-style behavior*) several behavioral change methods were used to increase autonomous motivation, future time perspective, optimism, and social support. Facilitators guide participants throughout the sessions using Motivational Interviewing (MI) techniques. MI is a client-centered, directive method that focuses on exploring and resolving ambivalence to change and appears to be particularly effective for individuals who are initially low in terms of readiness to change (40). MI supports a non-confrontational and supportive climate in which a non-judgmental, empathetic, and encouraging guidance is fostered to increase participants' intention to change health behavior (40).

After introductions, the first sessions starts with consciousness raising by showing a movie-clip about what a healthy life-style entails. Self-reevaluation was stimulated using scale questions about perceived own health and motivation to change, followed by the use of specific MI-techniques. Through reflective listening and positive affirmations, participants are stimulated to find more reasons for changing, increase their belief in the need to change and increase their expression of trust and change language to change their health behavior (40) (increasing autonomous motivation). Thereafter, personalizing risk was used by showing a second movie-clip about the positive and negative consequences of quitting (or not quitting) smoking that serves as an example for the following group discussion. Specific group exercises (matrix) in which participants reflect on the benefits and costs of both their current behavior and of changing that behavior using active and cooperative learning, stimulate to demonstrate more positive attributions about the benefits of changing their health behavior than not changing (increasing optimism) and their belief in the value of future health (increasing future time perspective). Performing these discussions in a group setting allows them to share experiences in order for them to serve as a role model for each other, which increases their belief that others will support reasons to improve health behavior (increasing social support).

After establishing a positive intention of participants to change their health behavior PO2 *(Employee with a low SEP sets at least 1 specific and achievable end goal to improve their life-style*) is targeted by using specific change methods to further increase their psychological capital, social support, and their autonomous motivation. Consciousness raising and chunking is used to inform participants (video-clip) about the effectiveness of, and how (in smaller steps) to set SMART (Specific, Measureable, Acceptable, Realistic, Time bound) goals. Thereafter, participants are encouraged to set one SMART health-related end-goal for themselves, with the help of specific exercises in their workbook, the facilitator, and group members, using active and cooperative learning. This increases participants' beliefs that SMART goal setting is effective in increasing their capability to change their health behavior in a sustainable way (increasing hope), and that they will be socially supported to change by for instance their colleagues (increasing social support). Furthermore, this increases their skills in setting SMART goals, boosting their confidence (efficacy) and providing them with an end-point to measure their success.

To achieve PO3 (*Employee with a low SEP divides the end-goal in at least 3 achievable, realistic and concrete sub goals*) another movie-clip about how to divide the end-goal in in incremental SMART sub-goals and complementary exercises in the work book were developed using chunking and setting graded tasks as change methods. These practical applications teach participants to demonstrate to divide their end-goal into at least 3 SMART sub-goals (increasing skills) and support their capability to change their health behavior in a sustainable way (increasing efficacy). The group setting using active and cooperative learning encourages processes whereby knowledge is created through the interpretation of experiences of the group members (direct experience). This increases participants' positive attributions about reaching the end-goal, their expressions of positive expectations about goal realization (increasing optimism), and their beliefs that relevant others will support their decision to improve their health (increasing social support).

To achieve PO4 *[Employee with a low SEP visualizes how end-goal can be reached and experiences and mentions (future) mental successes]*, several change methods are used. Goals visualization is used stimulating participants to think about their end-goal and the pathways and resources necessary to get there, leading to increased positive attributions about overcoming potential difficult situation (increasing efficacy and resilience) and awareness that their future health can be reached (increasing future time perspective). Furthermore, they are also stimulated to visualize celebrating small successes (positive cognitions) from reaching sub-goals (enactive mastery experiences).This reinforces participants' beliefs that they are capable in persevering toward their goals (increasing hope, efficacy, and resilience) and increases their optimism, feeding their motivation to change behavior. Active and cooperative learning and modeling is used to stimulate group discussion and collaboratively writing down how (small) future successes will be celebrated, which will increase feelings of being supported in the ability to reach goal(s) (increasing efficacy and social support).

For participants to realize PO5 *[Employee with a low SEP makes an inventory of obstacles and difficult situations he may encounter on the way to his goal(s)]* self re-evaluation, participatory problem solving and modeling were used as change methods. Specific exercises in the workbook, group discussions and the facilitator guide participants to make an inventory of obstacles and difficult situations to increase perseverance toward goals. Such an inventory encourages participants to use cognitive and affective assessments of one's self-image with and without an unhealthy behavior (self re-evaluations) increasing their awareness about putting effort in living more healthily now will result in better future health (future time perspective) and increasing their expectations and positive attributions about the effectiveness of making such an inventory (increasing hope and optimism). The group discussions aimed to share own or common obstacles to obtain feedback using participatory problem solving and function as role models to each other boosting participants confidence in their ability to make the inventory (increasing their efficacy and resilience) and increasing feelings of social support.

To achieve PO6 *[Employee with a low SEP formulates plans to overcome obstacles and difficult situations he may encounter on the way to his goal(s)]* active and cooperative learning and participatory problem solving are used in a group discussion where particular attention is paid to the obstacles one can encounter during goal attainment (resulting from PO5), and to ways how to overcome such obstacles, generating potential solutions, developing priorities, and making an action plan. A group discussion about the way setbacks might affect their motivation and sense of control over their behavior increases participants' awareness about the value of their future health (future time perspective), and stimulates positive expectations about their capability to persevere toward their goal(s) (increasing hope). Furthermore, it gives participants the opportunity to receive feedback from group members and the facilitator increasing their perceptions of social support. Enhancing network linkages is selected to encourage participants to identify who they pro-actively can ask for social support in their immediate environment—both in helping achieving the goal and coping with obstacles or setbacks. Research has shown that social support can positively influence the self-regulation of individuals increasing the likelihood of achieving goals (41).

Planning coping responses, implementation intentions and guided practice are used to prompt participants to list potential barriers and ways to overcome them, by linking situational cues with responses that are effective in attaining goals using “if-then..” plans (“if I encounter situation X, then I will engage in response Y” to reach the desired outcome). Participants are providing with a video-clip about how to make implementation intentions in several smaller steps (chunking) and are guided by their workbook, the participant group and the facilitator (guided practice), increasing participants' skills and efficacy to demonstrate making effective implementation intentions and increasing their confidence to actually overcome barriers or avoid difficulties on their way to their goal(s) (increasing efficacy and resilience) (42). Furthermore, planning coping responses and implementation intentions aim to increase participants' positive attributions and expectations about the effectiveness of making such plans and using effective coping strategies on their perseverance toward their goal(s) and their feelings of being in charge (increasing hope and optimism).

PO7 *[Employee with a low SEP adheres to the personally formulated plans to overcome or avoid obstacles and difficult situations on the way to the goal(s), monitors progress toward their goal(s) and celebrates successes of reached goals]*, relates to the aftercare process of the intervention. In this phase of the intervention, participants will be implementing their action plan for the next 12 weeks. The implementation intentions that are formulated in PO6 will now be implemented in real life situations and expect to increase participants' demonstration of overcoming or avoiding difficult situations and obstacles on their way to their sub-goals (increasing resilience) in such a way that they can also demonstrate reaching their sub-goals (increasing efficacy), which will increase their expectations of their capability in perseverance toward their end-goal (increasing hope).

Furthermore, participants will receive weekly reminders (prompts) that will stimulate them to monitor their progress and celebrate successes. Participants are encouraged to make a record (log) of their achievements in their workbooks to visualize their progress, increasing their autonomous motivation to change and maintain health-related behavior (43). This “coaching from the side-lines” has already shown to have a positive effect on achieving lifestyle-related goals and to increase PsyCap (44–46). Prompting self-monitoring of behavior increases participants' demonstration of monitoring their progress, their confidence in their ability to reach goals and the actual demonstration of reaching goal(s) (increasing efficacy). In response to this monitoring positive and constructive tailored feedback is given on a weekly basis (contingent rewards). This increases participants' feelings of being in charge of their own health (increasing hope), increases positive attributions about succeeding (increasing optimism), and their confidence to overcome difficulties and bounce back when encountering difficulties (increasing resilience). This approach will also allow participants' to re-evaluate themselves as, for example, individuals “that do not smoke,” making them aware that putting more effort into living healthier now, will result in a better future health (increasing future time perspective). Lastly, participants are stimulated to include their available social networks into their action plans (enhancing network linkages) aiming to increase participants' feelings of social support to overcome setbacks and persevere toward the end-goal.

### Program development and pre-testing (step 4)

#### Intervention materials

The theory- and evidence-based methods and applications (Step 3), the results of previous psychological capital interventions (44, 45), and of the needs assessment (Step 1) were combined and used to develop the actual intervention materials. Central to the intervention is the participant *workbook*. In the workbook, participants will keep note of their SMART formulated goal and sub-goals, how they aim to attain and celebrate them, the obstacles they may encounter and how to overcome them (their action plan), and a 12-week diary for monitoring their progress in the aftercare period. During the sessions, participants receive instructions and examples on how to use the workbook. For the SMART goals and implementation intentions, a step-by-step structure is provided in the workbook. The other intervention *materials* consist of a PowerPoint presentation used by facilitators to provide participants with visuals of the assignments and several video-clips during the intervention sessions, plasticized pictograms of health behaviors used during the intervention sessions, and a website (see www.futurepositive.nl) where the video-clips are accessible for participants (after login). In total, four video-clips were developed, using language and visual information that is easy to understand. The first video-clip informs participants about the intervention and can be used by organizations to facilitate recruitment. The two following video-clips provide information about a healthy life-style (video-clip 1) and about the advantaged and disadvantages of smoking cessation (and indirectly communicating the risks of not quitting smoking, video-clip 2). Video-clip 2 is shown to all participants, also the ones not focusing on smoking behavior, since it serves as an example how to think about pros and cons performing healthy or unhealthy behavior. The last two video-clips give participants tools how to define SMART goals and how they can make effective plans to cope with obstacles they may encounter on the way to their goals.

Results of the discussion session with the planning group where the intervention materials were presented indicated that the materials and the way the facilitator presented them were well-suited and appealing for the target group.

#### Results of pre-testing

Illustrative quotes resulting from the focus group discussions that were conducted to evaluate the pre-testing of the Future Positive micro-intervention are shown in Table 2. Experiences of the facilitator showed that recruitment of participants worked best when potential participants were addressed in a small group (e.g., during a regular work meeting), and in the presence of a supervisor or manager with a positive attitude toward the intervention. Recruitment efforts in larger work meetings or in the presence of supervisors/managers present who did not have a positive attitude toward the intervention were not successful. The focus group discussion indicated that the materials were appreciated and were understandable for the participants, the small group setting was appreciated, and the facilitator was evaluated as sufficiently emphasizing the autonomy of the participants, and having an open and non-judgmental attitude (basic attitude of motivational interviewing) that was specifically experienced as “pleasant” by participants. Most employees indicated in the focus group discussions that their motivation to change increased after the sessions and that this contributed to increasing their confidence to attain their goal. This may have improved participants hopefulness and optimism about their goal achievement. In addition, based on the way work books were filled out by participants, we may conclude that participants increased their skills in and demonstrated SMART goal setting in the sessions, as well as “if..then..” plans or how to avoid potentially difficult situations. Participants also demonstrated positive self-talk in the intervention sessions.

**Table 2:** Illustrative quotes from the focus group discussions.

**Illustrative quotes “Materials are easy to understand and are clearly presented in bite-sized chunks by the facilitator”**
“You (facilitator) explained well what I did not immediately understand“
“I liked the information, presented in the smoking video-clip”
“It's all very comprehensible”
“I thought it was interesting to see how long it took for nicotine to disappear from your body, that you actually saw improvement so quickly”
**Illustrative quotes “Materials appeal and participants recognize themselves in examples”**
“It's funny with those animations, it does appeal”
“I do recognize the situations”
**Illustrative quotes “Potential areas for improvement materials and sessions”**
“I think if it was a larger group you would have needed more time, because then someone might want to ask something extra or tell you something”
“I would have liked a little more time, especially making the plans”
**Illustrative quotes “Autonomy (part of motivational interviewing) is sufficiently conveyed by facilitator”**
“What I think is important is that we are not forced to change, that you can do it calmly, at your own pace”
“I like the fact that you can decide for yourself what you want to do”
**Illustrative quotes “increase in autonomic motivation”**
“It also stimulates, the assignments do make you think, and that stimulates me”
“Making such a plan is actually a good thing, because you also think about it, you are still working on it in your head”
“I've been saying for a long time, I want to stop, I want to stop, but this is more stimulating to actually do it”
”My motivation is now a 10, I'm looking forward to it. I'm glad I took part”
“I think it's a little more important now (after the sessions) to change”
“I am more motivated now” (after the workshop sessions)
**Illustrative quotes “increased psychological capital (efficacy, hope, resilience, and optimism)”**
“The confidence that I can do it too has become more” (First score 5 and now score 7 on scale question)
“The session makes me feel more confident now”
“Now I think more that I can actually do it”
**Illustrative quotes “Small group composition”**
“I really liked the small group setting”
**Illustrative quotes “Practicing with positive self-talk”**
“I'm going to make it this time”
“I'll just try again”

## Discussion

### Major findings and contributions to the field

Although health promotion efforts targeting the lifestyle-related behaviors among people with a low SEP may decrease socio-economic health disparities, this is a difficult to reach group (4), and the intervention materials used in most existing life-style enhancement programs are insufficiently suitable for people with a low SEP (5). To fill this gap in knowledge, the main aim of the current study was to systematically develop and describe the Future Positive micro-intervention, a micro-intervention that aims to increase life-style related health behaviors of employees with a low SEP. Intervention Mapping (IM), a step-wise participatory approach (8), was used to develop the program. Our main research questions during the first four steps of IM were: What are the needs of employees with a low SEP when it comes to their lifestyle-related behaviors; What should be changed to increase the lifestyle-related health behaviors of low SEP employees; How (what delivery context, mode and form) can the intervention be tailored to employees with a low SEP to effectively change their lifestyle-related behavior. First, a needs assessment was conducted to develop the logic model of the problem and to tailor the intervention to the needs of the target population. Second, the program goals were defined and the matrices of change, including performance and change objectives, were produced. Third, appropriate theory- and evidence-based methods and practical applications were selected. Last, a comprehensive program description was developed, including all intervention materials and the program was pre-tested in three groups of employees with a low socio-economic position. The final developed program is a micro-intervention consisting of two brief sessions and a 12 week aftercare period and will be delivered at the workplace in small groups guided by trained facilitators.

As was found in previously conducted studies (47), IM showed to be a useful tool to systematically develop a tailored intervention program. The insights into the psychological mechanisms of the intervention are described in detail, which can be used for future research and development. The detailed program description and developed materials will provide implementers of the intervention with the tools to effectively deliver the micro-intervention. Important findings regarding effective implementation of the program for organizations also emerged: To facilitate employees' participation, the intervention should be offered at the work place and during (paid) working hours. Furthermore, we also learned that recruitment efforts should focus on easy to understand information about what participation entails, as well as approaching employees in smaller groups in the presence of a supervisor/manager with a positive attitude toward the interventions (ambassador). These results are in line with findings from previous studies showing the importance of a low threshold character and understandable materials of interventions for people with a low SEP (48).

Qualitative results of the pre-testing showed that the intervention materials and setting were well-tailored to the target group: participants appreciated the materials, the fact that their autonomy was emphasized, and the small group setting. Furthermore, results indicated that participants experienced that their autonomous motivation to change and maintain healthier behavior was improved after following the intervention sessions, including their skills regarding SMART goal setting and applying implementation intentions. Based on the participants' experiences during pre-testing, participants also appeared to be more hopeful, optimistic and confident about their goal achievement. These preliminary results indicate that the intervention might increase PsyCap levels which is in line with previous research evaluating the effectiveness of PsyCap Interventions [e.g., (24–26)].

### Limitations and implications for future research and practice

One limitation of the current approach is our focus on the individual and on personal determinants of behavior. There is scientific evidence that environmental or contextual factors (e.g., adverse working conditions causing high job strain, or the neighborhood they live in) can contribute to (or limit) health promotion in this specific target group (48–51). However, it was not feasible to target these factors specifically by the planned intervention. Future studies should explore the preconditions for feasibility of integrating an effective environmental approach with the individual approach described by the Future Positive micro-intervention.

Furthermore, although the program is substantiated by theory and well-tailored to employees with a low SEP, future research still needs to show whether the intervention is effective in changing the health-behaviors in a proper effect evaluation (e.g., randomized-controlled trial). Insights of the qualitative results about how to approach and tailor intervention materials to people with a low SEP can be used for the development of other interventions targeting this group.

## Conclusions

The main aim of the current study was the systematic program development and description of the Future Positive micro-intervention, a micro-intervention that aims to increase life-style related health behaviors specifically developed for employees with a low SEP. The Future Positive micro-intervention has shown to be substantiated by theory, applicable in a work setting (high reach) and well-tailored to the needs of employees with a low SEP. The program is also likely to be suitable in different working contexts, since the pre-testing has been conducted in two different companies (cleaning and steel production industry) showing similar results. Furthermore, the findings of this study contribute to more knowledge about how to effectively reach this target population. This is important, since previous studies showed that most existing life-style enhancement programs fail to or insufficiently reach people with a low SEP (5). We therefore fill the gap in this existing range of interventions aimed to improve life-style behaviors and contribute to theory-based interventions aimed to decrease the SEP-Health gradient.

## Data availability statement

The datasets presented in this article are not readily available because some information in qualitative data collected in this study (personal in-depth interviews) may be linked to specific participants. Requests to access the datasets should be directed to karen.offermans@maastrichtuniversity.nl.

## Ethics statement

The studies involving human participants were reviewed and approved by Ethics Review Committee Psychology and Neuroscience (ERCPN) (Reference Number: 198_13_09_2018) Maastricht University. The patients/participants provided their written informed consent to participate in this study.

## Author contributions

KM and KS-O: study conception and design. KS-O: data collection and draft manuscript preparation. KS-O, KM, RR: analysis and interpretation of results. All authors reviewed the results, revised it critically for important intellectual content, and approved the final version of the manuscript.

## Funding

This study was funded by the Netherlands Organization for Health Research and Development (https://www.zonmw.nl/en/), Grant No. 531001410, awarded to KM. The funders had no role in the study design, data collection and analysis, decision to publish, or preparation of the manuscript.

## Conflict of interest

The authors declare that the research was conducted in the absence of any commercial or financial relationships that could be construed as a potential conflict of interest.

## Publisher's note

All claims expressed in this article are solely those of the authors and do not necessarily represent those of their affiliated organizations, or those of the publisher, the editors and the reviewers. Any product that may be evaluated in this article, or claim that may be made by its manufacturer, is not guaranteed or endorsed by the publisher.

## Supplementary material

Supplementary materials Acyclic behavioral change diagrams for the performance objectives of the future positive micro-intervention.
