# Supplementary material for: The Future Positive micro-intervention protocol: A program aiming to increase a healthy life-style among employees with a low socio-economic position
Source: Front Public Health. 2022 Sep 23;10:832447. doi: 10.3389/fpubh.2022.832447 (PMC9540196; doi:10.3389/fpubh.2022.832447)
Supplement: Supplementary materials — Acyclic behavioral change diagrams for the performance objectives of the future positive micro-intervention. [file Data_Sheet_1.ZIP › PO4.pdf]

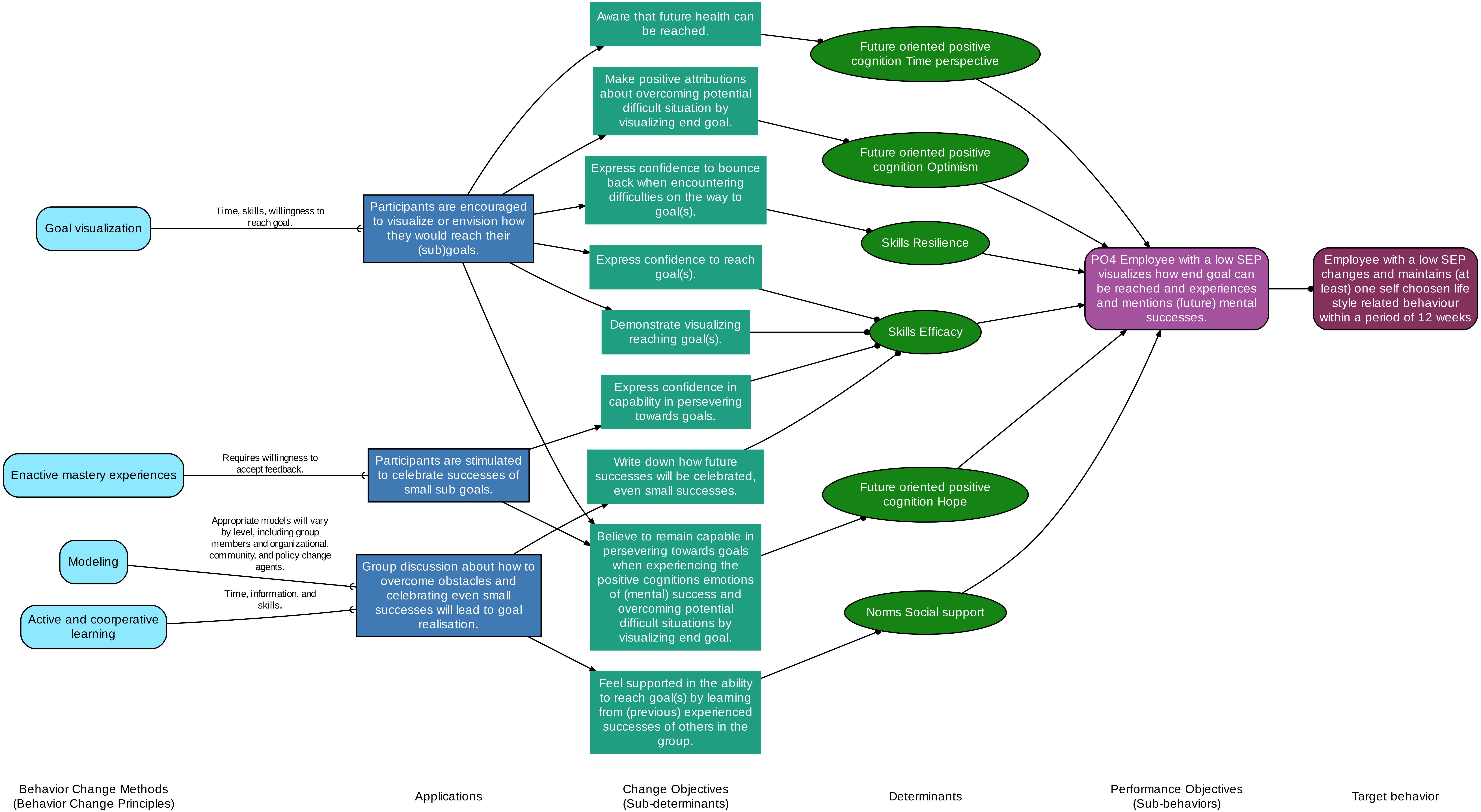
Goal visualization
Time, skills, willingness to reach goal.
Enactive mastery experiences
Requires willingness to accept feedback.
Modeling
Appropriate models will vary by level, including group members and organizational, community, and policy change agents.
Active and coorperative learning
Time, information, and skills.
Participants are encouraged to visualize or envision how they would reach their (sub)goals.
Participants are stimulated to celebrate successes of small sub goals.
Group discussion about how to overcome obstacles and celebrating even small successes will lead to goal realisation.
Aware that future health can be reached.
Make positive attributions about overcoming potential difficult situation by visualizing end goal.
Express confidence to bounce back when encountering difficulties on the way to goal(s).
Express confidence to reach goal(s).
Demonstrate visualizing reaching goal(s).
Express confidence in capability in persevering towards goals.
Write down how future successes will be celebrated, even small successes.
Believe to remain capable in persevering towards goals when experiencing the positive cognitions emotions of (mental) success and overcoming potential difficult situations by visualizing end goal.
Feel supported in the ability to reach goal(s) by learning from (previous) experienced successes of others in the group.
Future oriented positive cognition Time perspective
Future oriented positive cognition Optimism
Skills Resilience
Skills Efficacy
Future oriented positive cognition Hope
Norms Social support
PO4 Employee with a low SEP visualizes how end goal can be reached and experiences and mentions (future) mental successes.
Employee with a low SEP changes and maintains (at least) one self choosen life style related behaviour within a period of 12 weeks
Behavior Change Methods (Behavior Change Principles)
Applications
Change Objectives (Sub-determinants)
Determinants
Performance Objectives (Sub-behaviors)
Target behavior
