# Supplementary figures and images for: The Future Positive micro-intervention protocol: A program aiming to increase a healthy life-style among employees with a low socio-economic position
Source: Front Public Health. 2022 Sep 23;10:832447. doi: 10.3389/fpubh.2022.832447 (PMC9540196; doi:10.3389/fpubh.2022.832447)

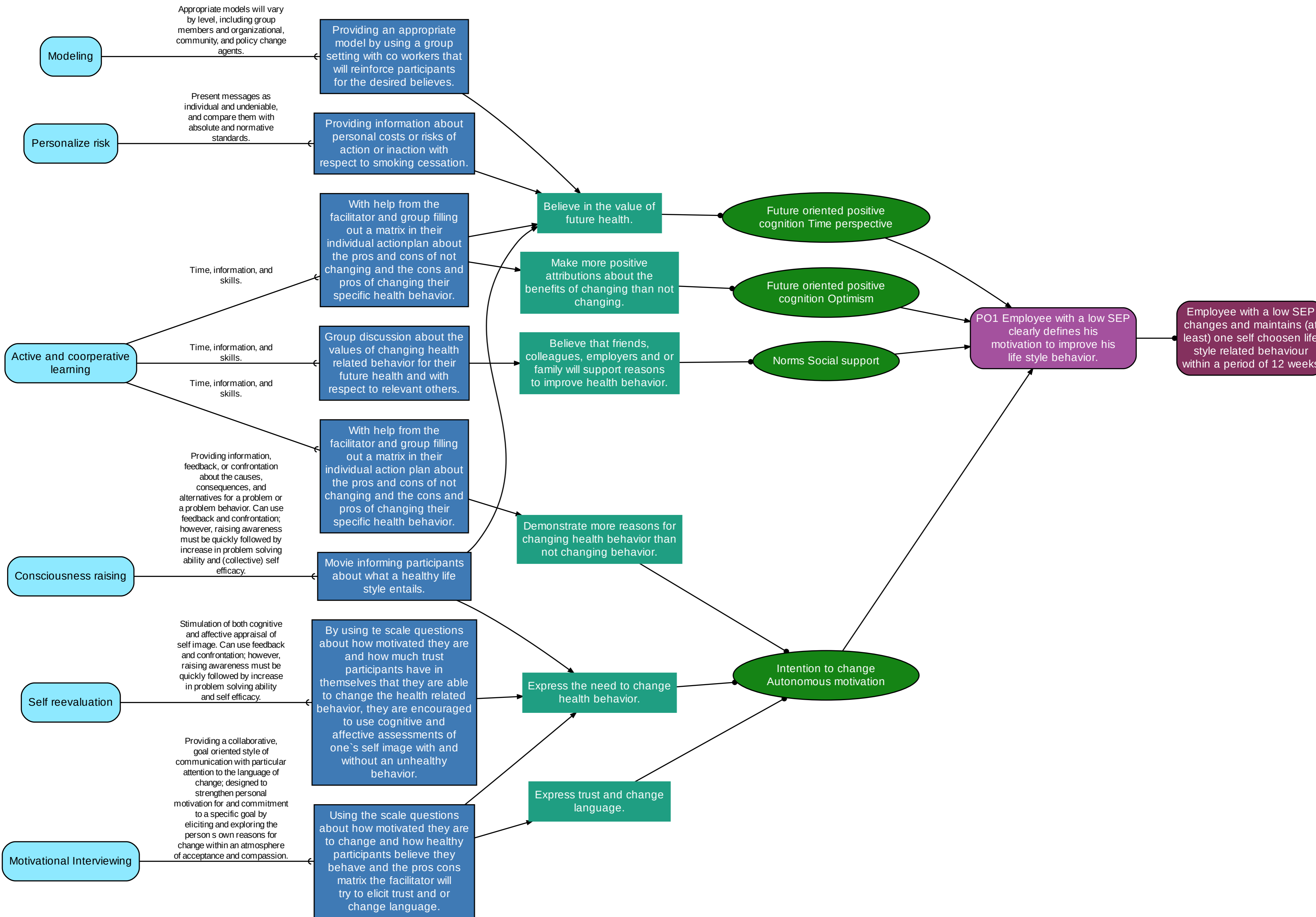

Supplement: Supplementary materials — Acyclic behavioral change diagrams for the performance objectives of the future positive micro-intervention. [file Data_Sheet_1.ZIP › PO1.pdf]

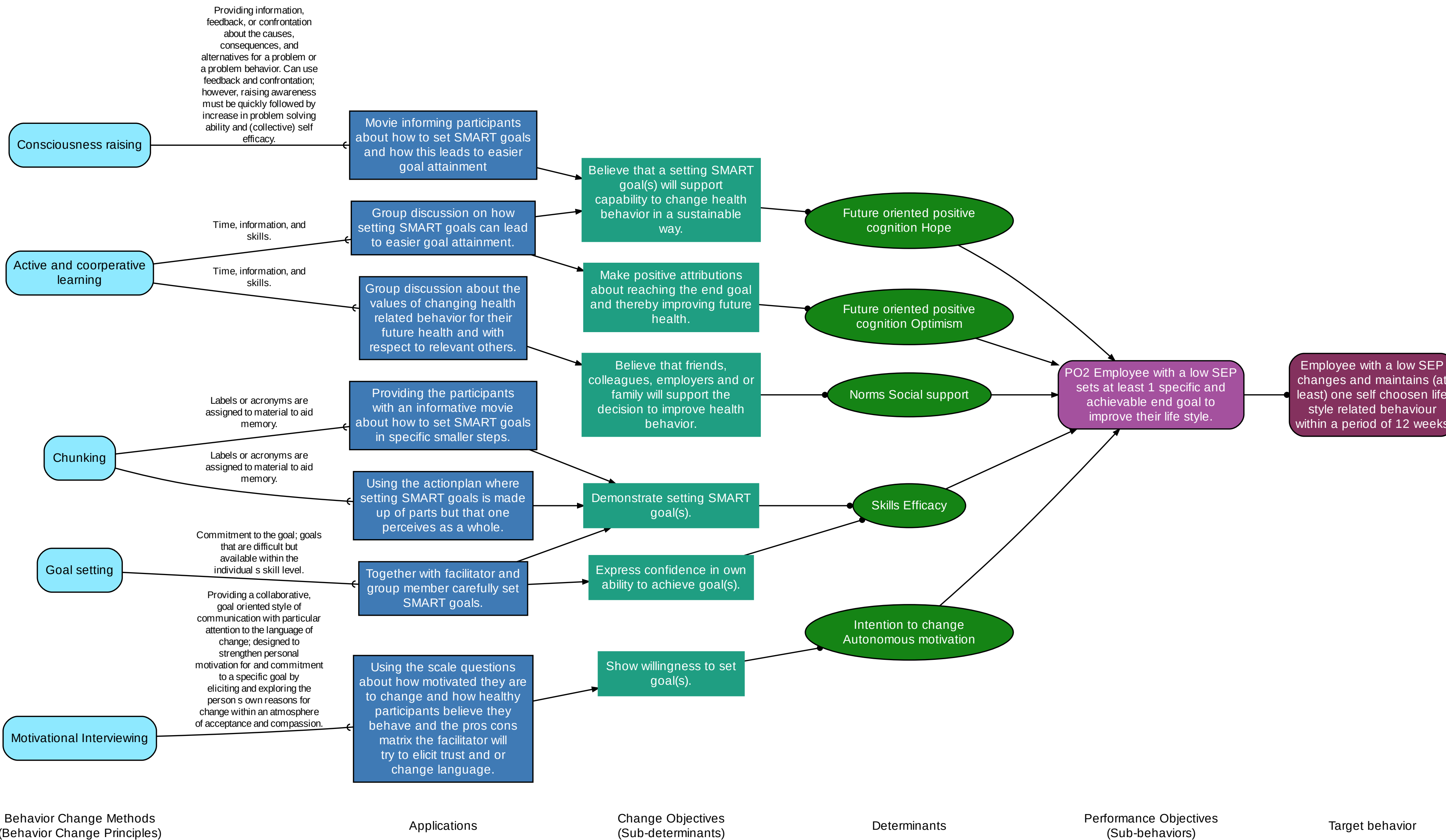

Supplement: Supplementary materials — Acyclic behavioral change diagrams for the performance objectives of the future positive micro-intervention. [file Data_Sheet_1.ZIP › PO2.pdf]

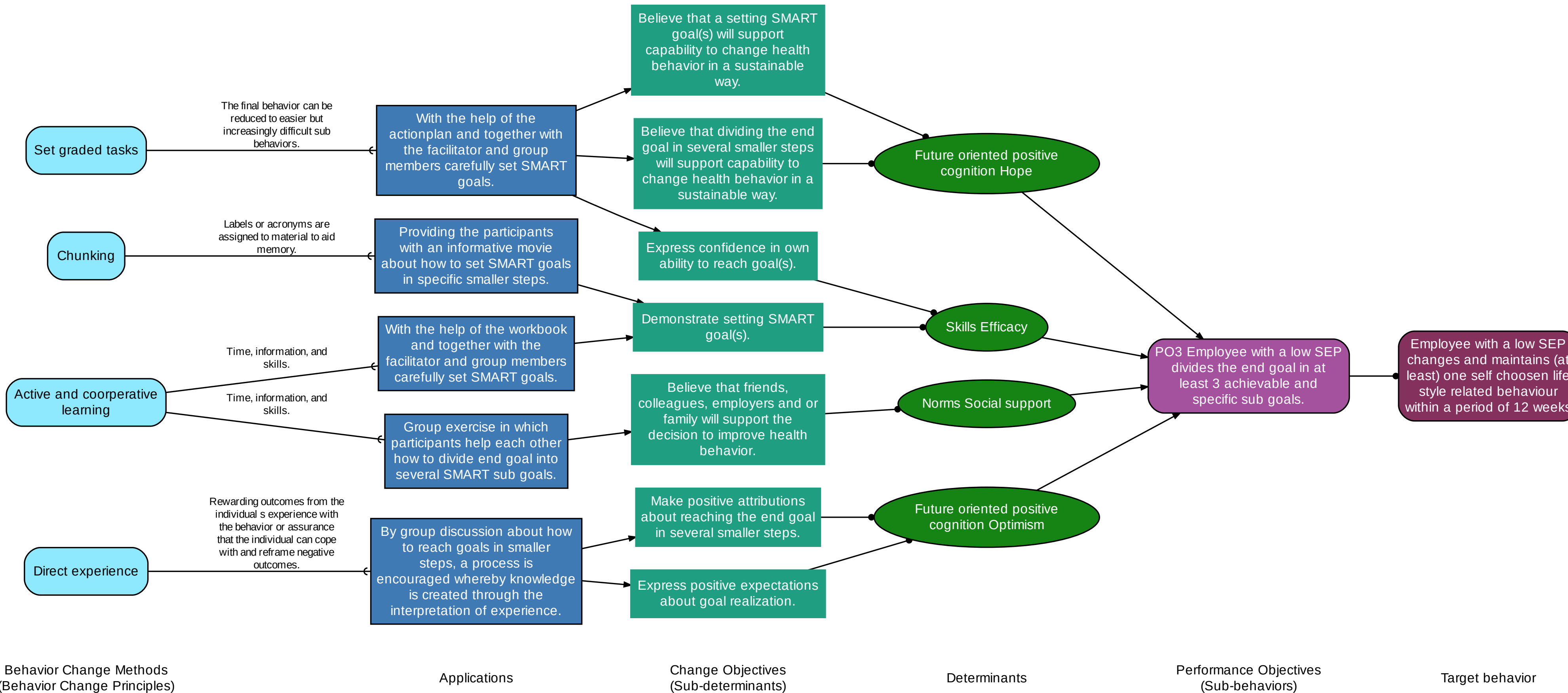

Supplement: Supplementary materials — Acyclic behavioral change diagrams for the performance objectives of the future positive micro-intervention. [file Data_Sheet_1.ZIP › PO3.pdf]

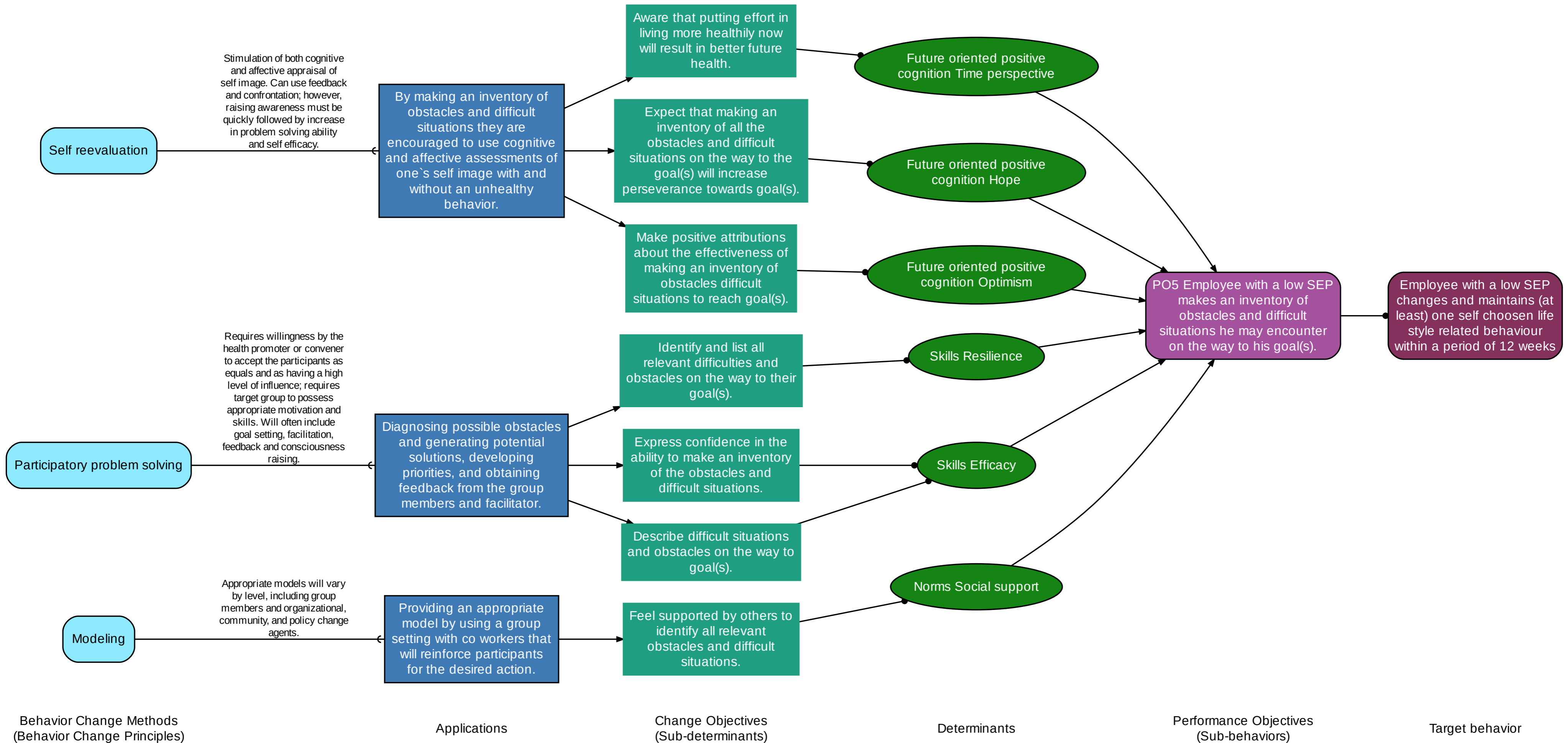

Supplement: Supplementary materials — Acyclic behavioral change diagrams for the performance objectives of the future positive micro-intervention. [file Data_Sheet_1.ZIP › PO5.pdf]

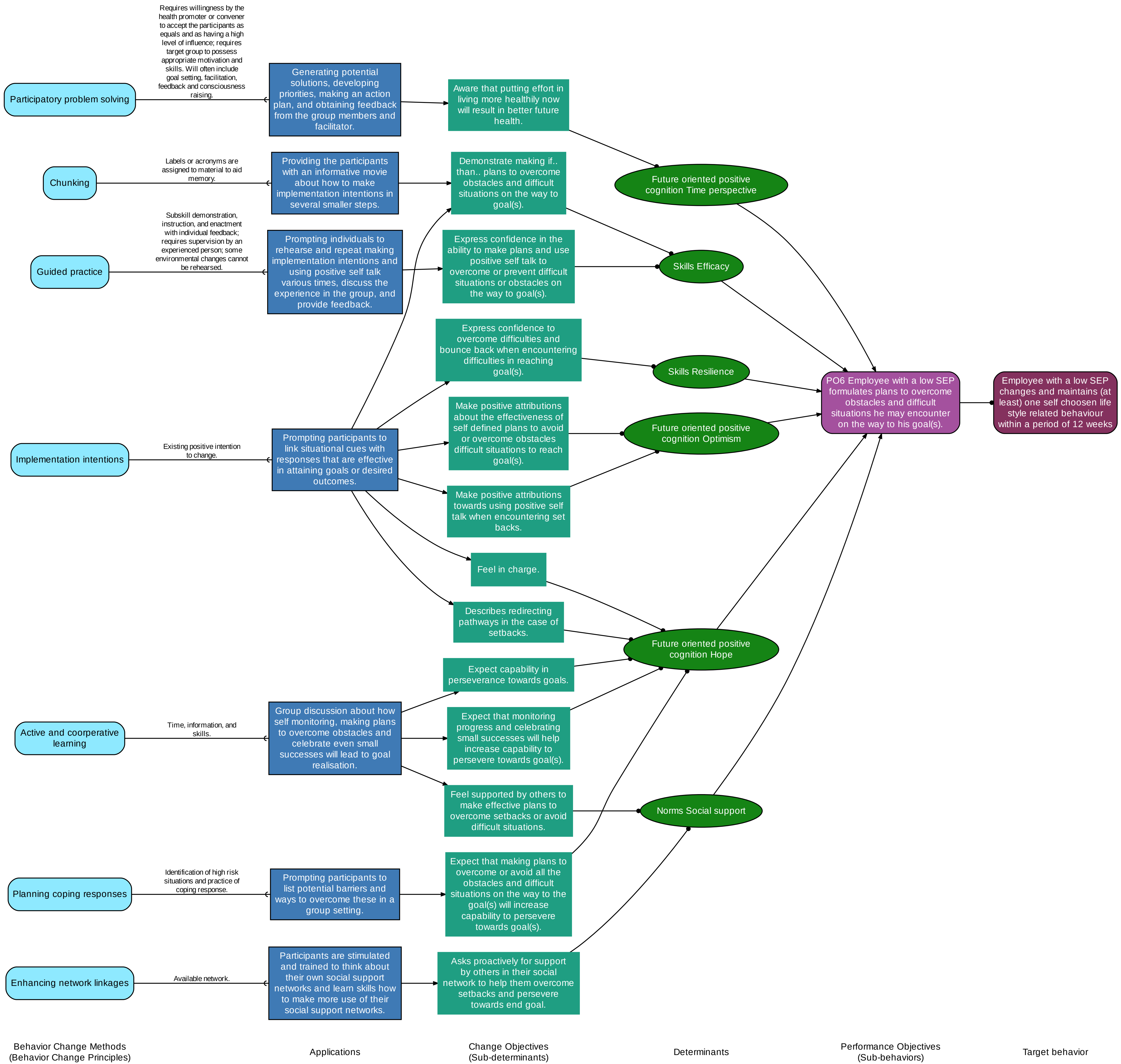

Supplement: Supplementary materials — Acyclic behavioral change diagrams for the performance objectives of the future positive micro-intervention. [file Data_Sheet_1.ZIP › PO6.pdf]

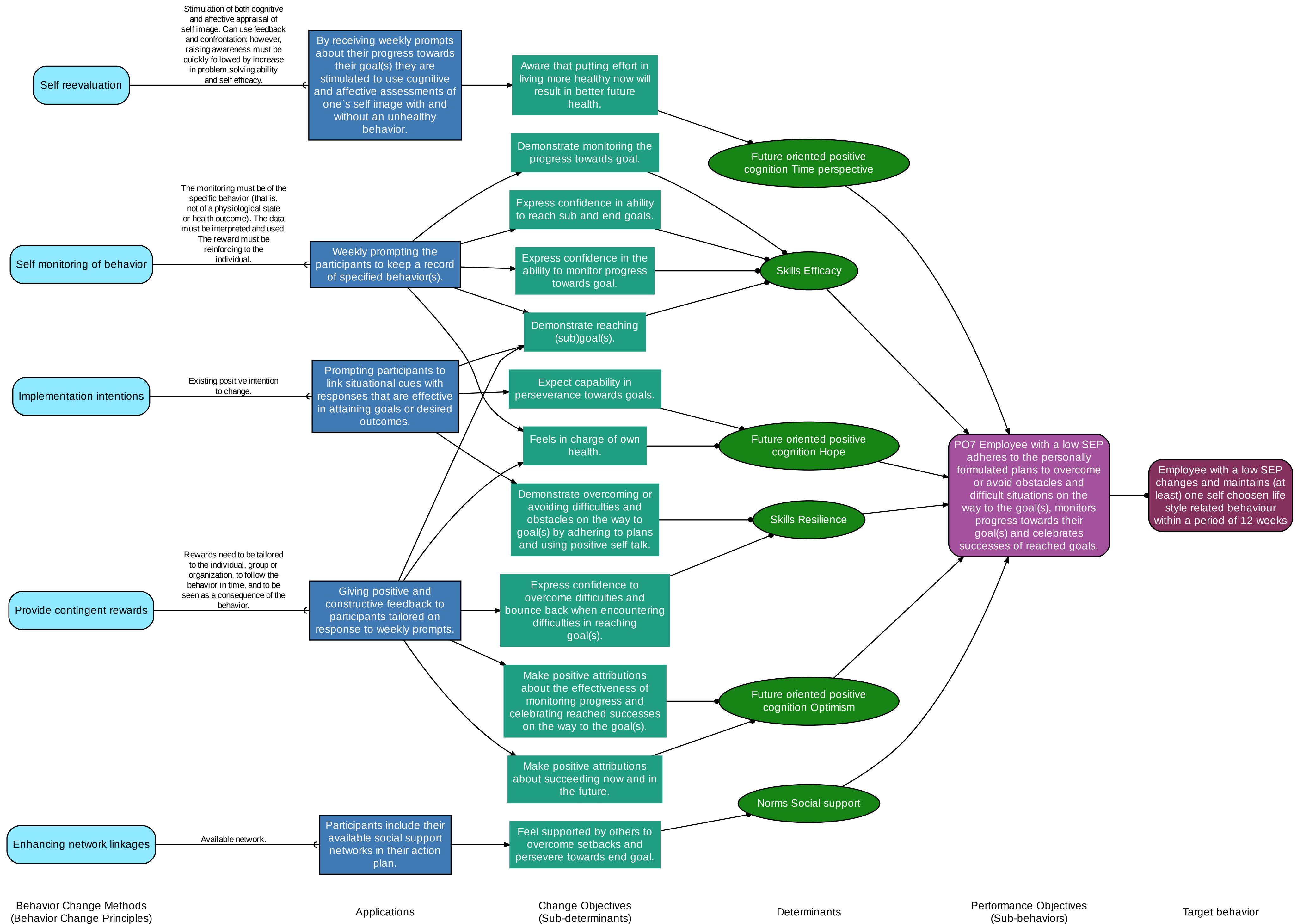

Supplement: Supplementary materials — Acyclic behavioral change diagrams for the performance objectives of the future positive micro-intervention. [file Data_Sheet_1.ZIP › PO7.pdf]
